# Abnormal cranium development in children and adolescents affected by syndromes or diseases associated with neurodysfunction

**DOI:** 10.1038/s41598-021-82511-x

**Published:** 2021-02-03

**Authors:** Agnieszka Guzik, Lidia Perenc, Mariusz Drużbicki, Justyna Podgórska-Bednarz

**Affiliations:** Department of Physiotherapy, Institute of Health Sciences, College of Medical Sciences, University of Rzeszów, Rzeszów, Poland

**Keywords:** Development of the nervous system, Diseases of the nervous system

## Abstract

Microcephaly and macrocephaly can be considered both cranial growth defects and clinical symptoms. There are two assessment criteria: one applied in dysmorphology and another conventionally used in clinical practice. The determination of which definition or under which paradigm the terminology should be applied can vary on a daily basis and from case to case as necessity dictates, as can defining the relationship between microcephaly or macrocephaly and syndromes or diseases associated with neurodysfunction. Thus, there is a need for standardization of the definition of microcephaly and macrocephaly. This study was designed to investigate associations between abnormal cranial development (head size) and diseases or syndromes linked to neurodysfunction based on essential data collected upon admission of patients to the Neurological Rehabilitation Ward for Children and Adolescents in Poland. The retrospective analysis involved 327 children and adolescents with medical conditions associated with neurodysfunction. Two assessment criteria were applied to identify subgroups of patients with microcephaly, normal head size, and macrocephaly: one system commonly used in clinical practice and another applied in dysmorphology. Based on the results, children and adolescents with syndromes or diseases associated with neurodysfunction present abnormal cranial development (head size), and microcephaly rarely co-occurs with neuromuscular disease. Macrocephaly frequently co-occurs with neural tube defects or neuromuscular diseases and rarely with cerebral palsy (p < 0.05); microcephaly frequently co-occurs with epilepsy and hypothyroidism (p < 0.001). Traditional classification facilitates the identification of a greater number of relationships and is therefore recommended for use in daily practice. There is a need to standardize the definition of microcephaly and macrocephaly and to include them in ‘Human Phenotype Ontology’ terms.

## Introduction

Microcephaly and macrocephaly are cranial growth defects as well as clinical symptoms. It is commonly known that head size is an indicator of brain size. The size of the cranium is assessed by measuring head circumference (HC), or more precisely occipitofrontal circumference (OFC), and by comparing the result to a biological reference system. Subsequently, it must be determined whether the head circumference measured is normal or abnormal, with the latter reflecting microcephaly or macrocephaly. There are two assessment criteria: one applied in dysmorphology and another traditionally used in clinical practice^1–6^. According to the first classification, microcephaly occurs when head circumference is smaller than the value corresponding to the difference between the mean and 3 standard deviations (x − 3SD), whereas macrocephaly occurs when the head circumference is larger than the value corresponding to the total of the mean and 3 standard deviations (x + 3SD). According to the second classification, microcephaly occurs when head circumference is smaller than the value corresponding to the difference between the mean and 2 standard deviations (x − 2SD) and macrocephaly when the head circumference is larger than the value corresponding to the total of the mean and 2 standard deviations (x + 2SD)^1–6^. As these definitions are not equivalent, an attempt has been made to identify which system should be used on a daily basis and the relationship between microcephaly or macrocephaly as well as the syndromes or diseases associated with neurodysfunction. Accordingly, there is a need for standardization of the definition of microcephaly and macrocephaly and for their inclusion in Human Phenotype Ontology (HPO) terms. Our own clinical observations have revealed that some of the children admitted to Neurological Rehabilitation Wards for Children and Adolescents in Poland are not diagnosed. We have sought to identify a relationship between symptoms and diagnosis in a group differentiated in terms of diagnosis: children and adolescents with congenital nervous system disorders or neurological syndromes with one or more neurodysfunctions visible since infancy. If a clear relationship between relative head size and diagnosis cannot be established, then knowledge of the relationship between microcephaly/macrocephaly and the group or subgroup of diseases with which it coexists may be a useful indicator in the differential diagnostic process.

This study was designed to investigate relationships between abnormal cranial development (head size) and diseases or syndromes linked to neurodysfunction based on essential data collected upon admission of patients to the Neurological Rehabilitation Ward for Children and Adolescents.

## Materials and methods

### Participants and setting

A retrospective study examined relevant information related to 327 patients receiving treatment during 2012–2016 at the Neurological Rehabilitation Ward for Children and Adolescents in St. Queen Jadwiga Regional Hospital No. 2 in Rzeszów, at the Clinical Regional Rehabilitation and Education Centre (KRORE), Poland. The following eligibility criteria were applied: age between 4 and 18 years; congenital nervous system disorder diagnosis or a neurological syndrome accompanied by at least one neurodysfunction manifesting from early childhood; availability of head circumference measurements; hospitalization in the period from 2012 to 2016; a single selected patient admission procedure; complete data (diagnosis, as well as anthropometric measurements—head circumference); and informed consent obtained from both the patient and parents/legal guardians. The following exclusion criteria were used: age under 4 or above 18 years (there was no biological frame of reference available); no diagnosis of a congenital nervous system disorder or neurological syndrome accompanied by at least one neurodysfunction manifesting in early childhood; more than one congenital nervous system disorder or neurological syndrome (for example: cerebral palsy (CP) co-occurring with Down syndrome; CP co-occurring with neural tube defect or with phenylketonuria); no head circumference measurements available; hospitalization before 2012 or after 2016; more than one selected patient admission procedure; lack of complete data (diagnosis, as well as anthropometric measurements); and lack of informed consent from both the patient and parents/legal guardians.

This study focuses only on classical definitions, taking into account the disturbance of the growth process (head circumference enlargement); the differentiation of body proportions (head size vs. body size) was not analyzed. Similar criteria were also adopted by Arroyo^4^, Sarno et al.^5^, and Jayaraman et al.^6^.

During the relevant period, 2637 children and adolescents were hospitalized at KRORE. An initial review of the medical records showed that 327 patients met the above inclusion criteria and that 2310 children did not meet the eligibility criteria. Ultimately, the retrospective analysis considered data related to 327 children (143 girls—43.7%, 184 boys—56.3%), with a mean age of 9.7 ± 4.3 years (median—9.0 years, minimum—4 years, maximum—18 years). Although there was a disproportionate ratio of boys to girls in our cohort, many similar studies enrolling more boys than girls have been conducted in the field of neurodysfunction. It was therefore concluded that conducting such a research analysis of this type would be acceptable given the data available^7,8^.

### Study protocol

The study was reviewed and approved by the Bioethics Commission of the Medical Faculty at the University of Rzeszow (Approval Ref. no. 10/04/2019). All procedures were carried out in full compliance with the Declaration of Helsinki. Informed consent was given by the children and their parents/legal guardians and by the director of the hospital. Letters of consent were received before the study protocol was filed with the bioethics committee.

### Procedures and data analyses

Basic data, acquired upon the patients’ admission, i.e., age, sex, principal and additional diagnosis, and head circumference (HC), were considered in the retrospective analysis. Anthropometric measurements were performed by KRORE staff in accordance with the principles adopted in the hospital; principal and additional diagnoses, as determined prior to hospitalization at KRORE, were performed by relevant specialists (geneticists, neurologists, endocrinologists, etc.). The patients involved in the study presented various diseases or syndromes linked with nervous system damage; more specifically, they were all affected by congenital disorders and/or conditions, with or without encephalopathy, in association with motor defects (neurodysfunctions) manifesting from infancy. The patients were divided into subgroups according to the suspected presence or lack of encephalopathy, etiopathogenesis and nature, following the criteria proposed in the literature^1^. The intention of the authors was to indicate the relationship with the disease or syndrome, and if impossible, based on different subgroups. This would allow for an effective differential diagnostic process with a given subgroup of diseases and/or syndromes^1,3^.

To identify subgroups of patients with microcephaly, normal head size, and macrocephaly, two assessment criteria were applied: one system commonly used in clinical practice and another applied in dysmorphology^2,4–6^. For analyses of all of the above criteria, the z-score for head circumference (z-score HC) was computed for each patient. Normative values published earlier^9^ were applied as references. For years, one of the authors has been participating in research on the secular trend in the development of children and youth from Rzeszow (the capital of Podkarpackie Province). Research on the somatic development of children and adolescents from Rzeszow began in 1978/1979 and was repeated in 1993/1994, 2003/2004 and 2013/2014^9–15^, which enabled ascertainment of the rules of growth in children and adolescents. At the Clinical Regional Rehabilitation and Education Centre (Rzeszow, Poland), the daily clinical work of this author involves children and adolescents with neurodysfunctions, and she often observes changes in their physical development. To define disorders such as microcephaly/macrocephaly, an additional anthropometric parameter (head circumference) was introduced during the research conducted in 2013/2014^9^. It was the first time that tables of norms containing mean values and standard deviation of the head circumference, taking into account age and sex, were presented^9^. Both studied groups of school children (Rzeszow, Poland) and the Clinical Regional Rehabilitation and Education Centre (Rzeszow, Poland) are ethnically homogeneous. A similar methodology based on the z-score and reference system was used earlier in research^16^. When calculating the z-score, the value of the anthropometric feature in children and adolescents affected by syndromes or diseases associated with neurodysfunction was used, as was the value of the mean and standard deviation of the anthropometric feature from the reference system, taking into account sex and age^16^. The selected anthropometric parameter used was head circumference^9^.

Further analyses focused on possible correlations between co-occurring abnormalities of cranial development, i.e., microcephaly or macrocephaly, and medical conditions linked to neurodysfunction; assessment relative to the subgroups identified and within these subgroups was also carried out. Furthermore, additional diagnoses, i.e., hypothyroidism, symptomatic epilepsy and genetically conditioned epilepsy syndrome were considered. Adjusted standardized residuals (ASRs) are shown in crosstabs, in addition to percentages. Values below and above the threshold of 1.96 were assumed to reflect a smaller and a greater number, respectively, than in the case of a random distribution. Methods of statistical inference were used to assess to what degree intergroup differences corresponded to certain regularities found in the target population and to what extent this could be due to some random differences. A chi-square test of independence was applied due to the nominal nature of the characteristics subjected to comparative analyses. Relationships between the dependent qualitative and independent quantitative variables were examined using nominal regression. Statistical significance was assumed at p < 0.05.

## Results

Seven subgroups were identified (Table 1A). Six of these comprised children with medical conditions usually presenting with encephalopathy, as follows: progressive metabolic disorders (2.1%), progressive epilepsy—genetically determined epileptic syndromes (0.3%), nonprogressive cerebral palsy (73.1%), nonprogressive neural tube defects (7.3%), nonprogressive genetic disorders (chromosomal aberrations, monogenic disorders excluding neuromuscular diseases) (7%), and nonprogressive toxicity (0.3%). There was also one subgroup comprising patients with neuromuscular disorders (9.8%), which commonly occur without encephalopathy. Based on the expected presence and characteristics of encephalopathy^1,3^, the six small subgroups were combined into two larger subgroups corresponding to progressive encephalopathy (PE) (2.4%) and nonprogressive encephalopathy (NPE) (88.1%); the third group consisted of patients with neuromuscular diseases (NMDs) (9.8%). Given that neural tube defects vary greatly^17^ and that surgical treatment is of key importance for development^18^, the children in this subgroup were divided based on the cause of the surgery (myelomeningocele and hydrocephalus versus myelomeningocele alone). The remaining cases, for which no surgical treatment was applied, are also presented. Both chromosomal aberrations and genetic mutations were included in the subgroup with genetic disorders (GD), as in other studies^19,20^. The characteristics of the study group are presented in Table 1.

**Table 1:** Characteristics of the study group (A) and abnormal cranial development (B–D).

(A) Characteristics of 327 patients with neurodysfunction								
Diseases and syndromes associated with neurodysfunction (principal diagnosis)			Classification with regard to the etiopathogenesis, presence and character of encephalopathy			Classification with regard to the presence and character of encephalopathy		
	N	%*		N	%*		N	%
NBIA-MPAN, Neurodegeneration with Brain Iron Accumulation—Mitochondrial Protein Associated Neurodegeneration	2	0.6	MD, encephalopathy in metabolic disorder	7	2.1	PE, progressive encephalopathy	8	2.4
GSD II, Pompe disease	1	0.3						
LCHAD, long-chain 3-hydroxyacyl-coenzyme A dehydrogenase deficiency	1	0.3						
SLO, Smith–Lemli–Opitz syndrome	1	0.3						
GLUT1d, glucose transporter 1 deficiency	1	0.3						
NKH, nonketotic hyperglycinemia	1	0.3						
SMEI, Dravet syndrome	1	0.3	EE, epileptic encephalopathy	1	0.3			
sasMMC&HCP, state following surgery due to lumbar myelomeningocele and hydrocephalus	17	5.2	NTDs, encephalopathy in neural tube defects	24	7.3	NPE, nonprogressive encephalopathy	287	88.1
sasMMC, state following surgery due to lumbar myelomeningocele	3	0.9						
sasMM, state following surgery due to parieto-occipital meningocele	1	0.3						
ACM, Arnold–Chiari malformation	2	0.6						
HCP, isolated hydrocephalus	1	0.3						
DS, Down syndrome	11	3.4	GD, encephalopathy in genetic disorders	23	7.0			
ES, Edwards syndrome	1	0.3						
PMS, Phelan–McDermid syndrome	2	0.6						
MWS, Mowat–Wilson syndrome	1	0.3						
AS, Angelman syndrome	1	0.3						
DGS, Di George syndrome	1	0.3						
46,XY,del(X)(q24)	1	0.3						
CdLS, Cornelia de Lange syndrome	1	0.3						
SDS, Schwachman–Diamond syndrome	1	0.3						
PWS, Prader–Willi syndrome	1	0.3						
46 XX, add(2)(q25)	1	0.3						
46XX, del (12) (q24.21q24.23)	1	0.3						
FAS, fetal alcohol syndrome	1	0.3	TE, toxic encephalopathy	1	0.3			
CP, cerebral palsy	239	73.1	CP encephalopathy in cerebral palsy	239	73.1			
HMSN, hereditary motor and sensory polyneuropathy	8	2.4	NMD, neuromuscular disorders	32	9.8	NMD, neuromuscular disorders	32	9.8
LGMD, muscular dystrophy limb-girdle	7	2.1						
BMD, Becker muscular dystrophy	3	0.9						
DMD, Duchenne muscular dystrophy	7	2.1						
TD, Thomsen disease	1	0.3						
AMC&N arthrogryposis multiplex congenita with neuropathy	3	0.9						
CM, congenital myopathy	1	0.3						
SMA, spinal muscular atrophy	2	0.6						
Total	327	99,7	Total	327	99,9	Total	327	100

(B) Numerical characteristics of z-score HC								
z-score	*N*	$\overline{x}$	Me	*SD*	*c*_25_	*c*_75_	Min	Max
z-score HC	327	− 0.53	− 0.54	2.14	− 1.71	0.86	− 7.36	8.29

(C) Cranial growth abnormalities (the size of the head)—criteria								
The size of the head	Dysmorphology classification (HC)	Traditional classification (HC)						
Normal	− 3 ≥ z-score HC ≤ 3	− 2 ≥ z-score HC ≤ 2						
Microcephaly	z-score HC < − 3	z-score HC < − 2						
Macrocephaly	z-score HC > 3	z-score HC > 2						

(D) Prevalence of cranial growth abnormalities (the size of the head)								
Dysmorphology classification (HC)		The size of the head	Traditional classification (HC)					
N	N%		N	N%				
273	83.5	Normal	224	68.5				
41	12.5	Microcephaly	72	22				
13	4.0	Macrocephaly	31	9.5				

*N* numbers of patients, % percent, *z-score HC* z-score for head circumference, *SD* standard deviation, *Min* minimum value, *Max* maximum value.

^a^The percentages have been rounded to one digit after the decimal point, so the total value is 99.7% and 99.9% instead of 100%

Patients with CP constituted a significant majority (73.1%). Based on the diagnoses, the cases involved spastic (93.7%; N = 223), mixed (5%; N = 12), and ataxic (1.7%; N = 4) types of CP, as based on Hagberg’s classification^1,3^; no cases involved a dyskinetic type. In the group of children with spastic CP, 40.4% were found to have diplegia (N = 90), 34.1% (N = 76) to have tetraplegia, and 25.6% to have hemiplegia (N = 57). The primary diagnoses were accompanied by additional diagnoses of symptomatic epilepsy 26.3% (N = 86) and hypothyroidism 4.3% (N = 14). Selected numerical characteristics of z-score HC, i.e., the arithmetic mean ($\overline{x}$), median (Me), standard deviation (SD), minimum (Min), maximum value (Max), 25th centile (*c*_25_) and 75th centile (*c*_75_), are shown in Table 1B. The mean and median z-score HC in the study group was lower than 0 and higher than − 1, respectively.

Taking into account dysmorphology criteria (Table 1C), normal head size was found in 83.5% of the patients, as compared to 16.5% of the patients presenting with abnormalities, namely, microcephaly 12.5% and macrocephaly 4.0% (Table 1D). In accordance with traditional criteria (Table 1C), normal head size was found in 68.5%, and abnormalities were identified in 31.5% of the patients (microcephaly 22% and macrocephaly 9.5%) (Table 1D).

Subsequent analyses focused on relationships between co-occurring abnormalities of cranial growth (head size) and diseases/syndromes associated with neurodysfunction, also relative to (and between) the identified subgroups. Additional diagnoses were also considered.

**Figure 1:**
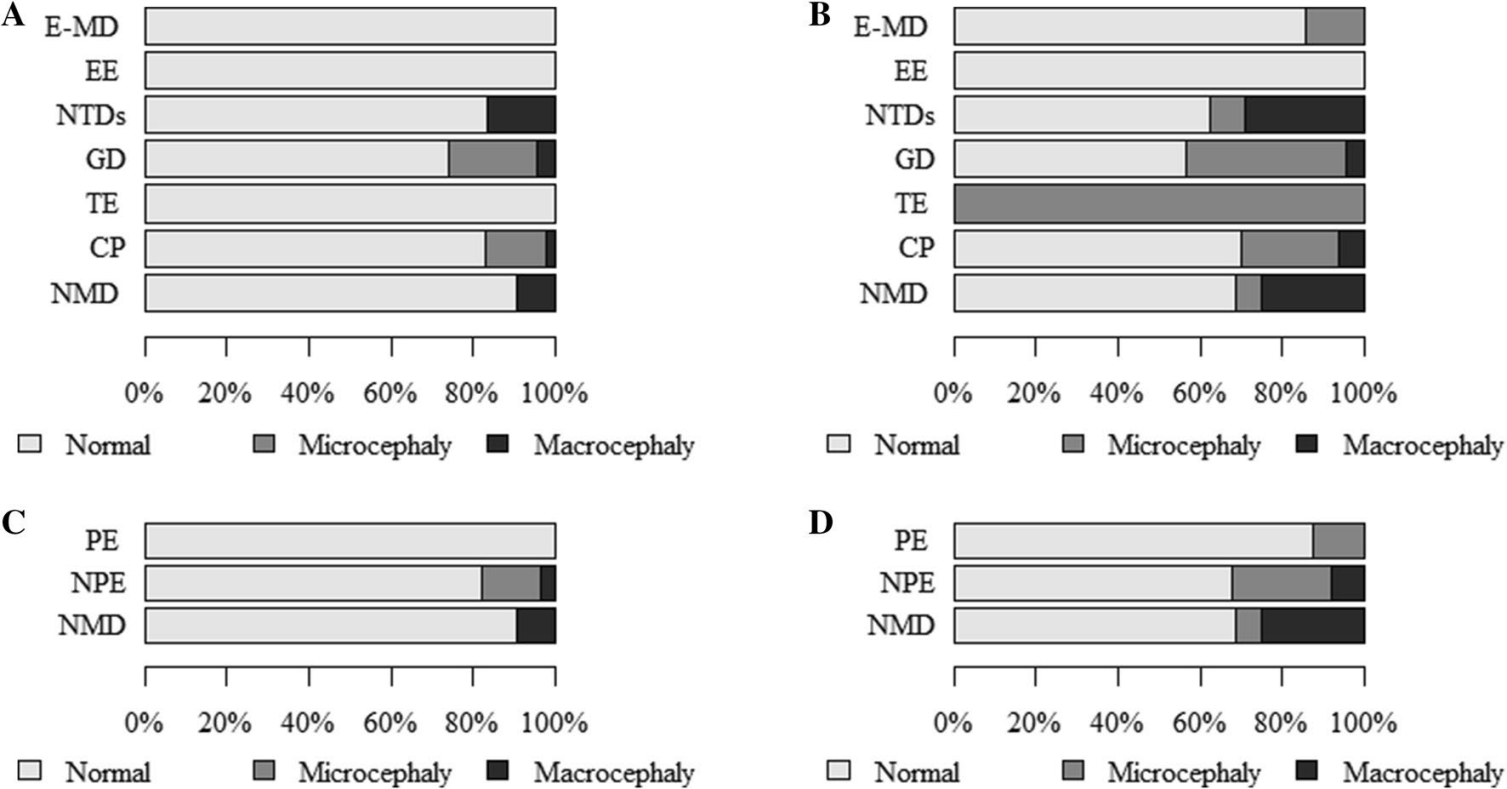
The size of the head: (**A**)—dysmorphology classification and classification based on the etiopathogenesis, presence and character of encephalopathy; (**B**)—traditional classification and classification based on the etiopathogenesis, presence and character of encephalopathy; (**C**)—dysmorphology classification and classification based on the presence and character of encephalopathy; (**D**)—traditional classification and classification based on the presence and character of encephalopathy.

**Figure 2:**
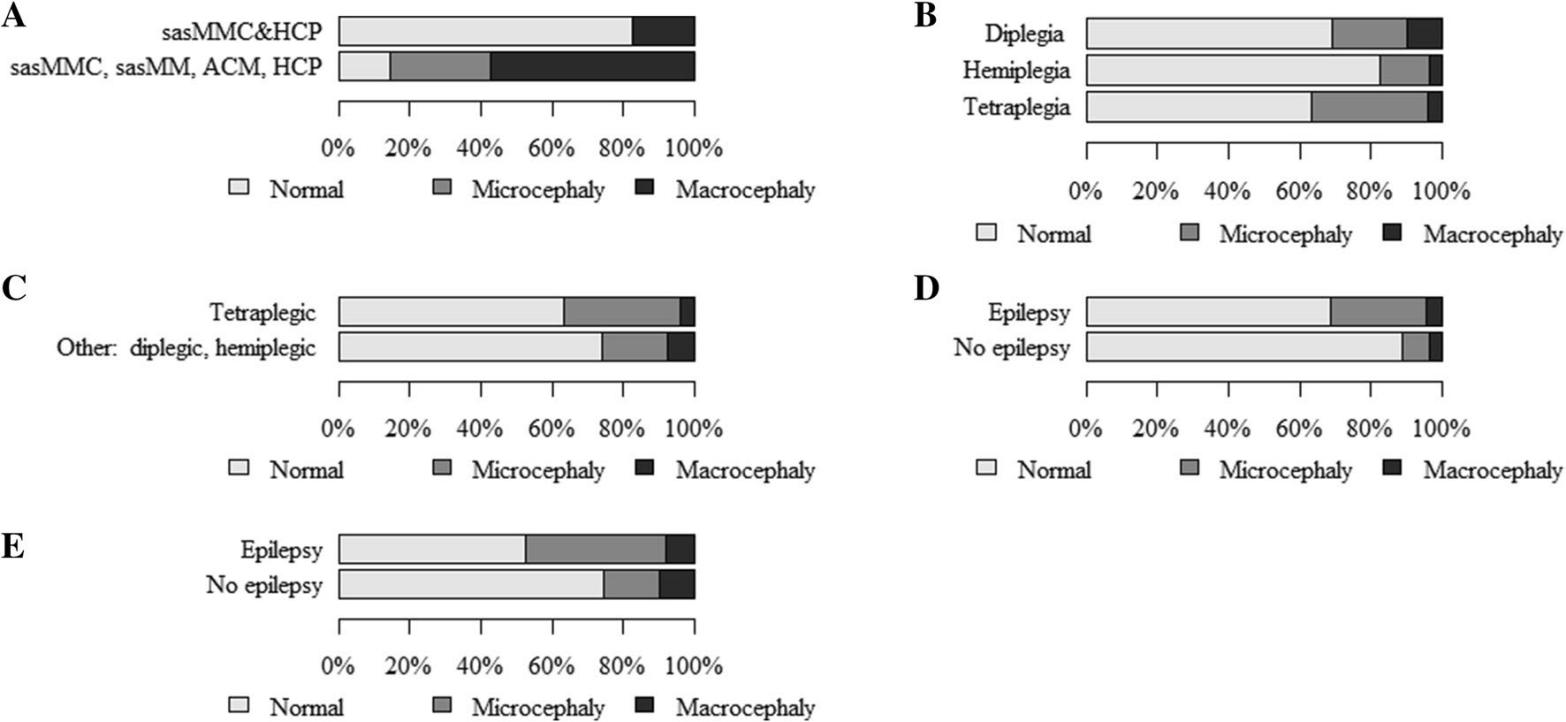
The size of the head: (**A**)—traditional classification and encephalopathy in neural tube defects; (**B**)—traditional classification and type of spasticity (Diplegia, Hemiplegia, Tetraplegia); (**C**)—traditional classification and type of spasticity (Tetraplegic, Other: diplegic and hemiplegic together); (**D**)—dysmorphology classification and epilepsy; (**E**)—traditional classification and epilepsy.

**Figure 3:**
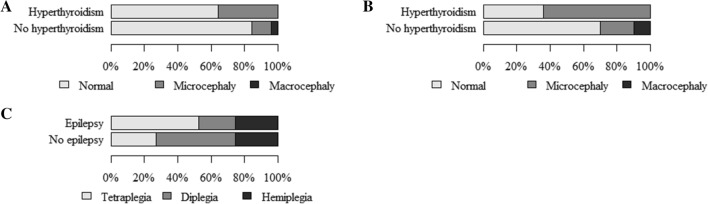
The size of the head: (**A**)—dysmorphology classification and hypothyroidism; (**B**)—traditional classification and hypothyroidism; (**C**)—relationship between epilepsy and type of spasticity.

The findings showed more/less common co-occurrence of as well as statistically significant relationships between *the size of the head—dysmorphology classification (HC) and the following*:

- entities/syndromes associated with neurodysfunction (Table 2A). Normal head size rarely co-occurred with isolated hydrocephalus (HCP), Edwards syndrome (ES), or Di George syndrome (DGS). Microcephaly frequently co-occurred with ES, DGS, and CP. Macrocephaly was rarely found to co-occur with CP and frequently with ACM (Arnold–Chiari malformation), HCP, Phelan–McDermid syndrome (PMS), and Becker’s muscular dystrophy (BMD). The relationship was statistically significant (p = 0.026),
- classification with regard to the etiopathogenesis, presence and character of encephalopathy (Table 2B and Fig. 1A). Microcephaly was found to co-occur more frequently in children with CP but rarely in those with NMD. Macrocephaly frequently co-occurred with neural tube defects (NTDs) but rarely with CP. The relationship was statistically significant (p = 0.010),
- classification with regard to the presence and character of encephalopathy (Table 4A and Fig. 1C). Microcephaly frequently occurred in NPE and rarely in the subgroup with NMD. The relationship was statistically significant (p = 0.060, Cp = 0.146),
- epilepsy (Table 4F and Fig. 2D). Normal head size rarely co-occurred with epilepsy and frequently with a lack of epilepsy. Microcephaly and epilepsy were often found to co-occur. Microcephaly and lack of epilepsy co-occurred rarely. The relationship was statistically significant (p < 0.001),
- hypothyroidism (Table 5A and Fig. 3A). Normal head circumference often co-occurred with a lack of hypothyroidism and was rarely found in patients with hypothyroidism. Microcephaly frequently co-occurred with hypothyroidism and rarely in the absence of this condition. The relationship was statistically significant (p = 0.024, Cp = 0.105).

**Table 2:** The size of the head—dysmorphological classification (HC) and (A) units and syndromes associated with neurodysfunction, (B) classification with regard to the etiopathogenesis, presence and character of encephalopathy.

(A) The size of the head—dysmorphology classification (HC) and entities/syndromes associated with neurodysfunction							
Entities and syndromes associated with neurodysfunction	The size of the head—dysmorphology classification (HC) by z-score HC (*p* = 0.026; Cp = 0.465)						Total
	Normal		Microcephaly		Macrocephaly		
	N (%)	ASR	N (%)	ASR	N (%)	ASR	N (%)
NBIA-MPAN	2	0.6	0	− 0.5	0	− 0.3	2
GSD II	1	0.4	0	− 0.4	0	− 0.2	1
LCHAD	1	0.4	0	− 0.4	0	− 0.2	1
SLO	1	0.4	0	− 0.4	0	− 0.2	1
GLUT1d	1	0,4	0	− 0.4	0	− 0.2	1
NKH	1	0.4	0	− 0.4	0	− 0.2	1
SMEI	1	0.4	0	− 0.4	0	− 0.2	1
sasMMC&HCP	15 (88%)	0.5	0	− 1.6	2 (12%)	1.7	17
sasMMC	3	0.8	0	− 0.7	0	− 0.4	3
sasMM	1	0.4	0	− 0.4	0	− 0.2	1
ACM	1 (50%)	− 1.3	0	− 0.5	1 (50%)	3.3	2
HCP	0	− 2.3	0	− 0.4	1	4.9	1
DS	8 (73%)	− 1.0	3 (27%)	1.5	0	− 0.7	11
ES	0	− 2.3	1	2.6	0	− 0.2	1
PMS	1 (50%)	− 1.3	0	− 0.5	1 (50%)	3.3	2
MWS	1	0.4	0	− 0.4	0	− 0.2	1
AS	1	0.4	0	− 0.4	0	− 0.2	1
DGS	0	− 2.3	1	2.6	0	− 0.2	1
46,XY,del(X)(q24)	1	0.4	0	− 0.4	0	− 0.2	1
CdLS	1	0.4	0	− 0.4	0	− 0.2	1
SDS	1	0.4	0	− 0.4	0	− 0.2	1
PWS	1	0.4	0	− 0.4	0	− 0.2	1
46 XX, add(2)(q25)	1	0.4	0	− 0.4	0	− 0.2	1
46XX, del (12) (q24.21q24.23)	1	0.4	0	− 0.4	0	− 0.2	1
FAS	1	0.4	0	− 0.4	0	− 0.2	1
CP	198 (83%)	− 0.5	36 (15%)	2.3	5 (2%)	− 2.9	239
HMSN	7 (87.5%)	0.3	0	− 1.1	1 (12.5%)	1.2	8
LGMD	7	1.2	0	− 1.0	0	− 0.5	7
BMD	2 (67%)	− 0.8	0	− 0.7	1 (33%)	2.6	3
DMD	6 (86%)	0.2	0	− 0.4	1 (14%)	1.4	7
TD	1	0.4	0	− 0.4	0	− 0.2	1
AMC&N	3	0.8	0	− 0.7	0	− 0.4	3
CM	1	0.4	0	− 0.4	0	− 0.2	1
SMA	2	0.6	0	− 0.5	0	− 0.3	2
Total	273 (83.5%)		41 (12.5%)		13 (4%)		327

(B) The size of the head—dysmorphological classification (HC) and classification with regard to etiopathogenesis, presence and character of encephalopathy							
Classification with regard to the etiopathogenesis, presence and character of encephalopathy	The size of the head—dysmorphology classification (HC) by z-score HC (*p* = 0.010; Cp = 0.273)						
	Normal		Microcephaly		Macrocephaly		Total
	N (%)	ASR	N (%)	ASR	N (%)	ASR	N (%)
E-MD	7	1.2	0	− 1.0	0	− 0.5	7
EE	1	0.4	0	− 0.4	0	− 0.2	1
NTDs	20 (83%)	0.0	0	− 1.9	4 (17%)	**3.3**	24
GD	17 (74%)	− 1.3	5 (22%)	1.4	1 (4%)	0.1	23
TE	1	0.4	0	− 0.4	0	− 0.2	1
CP	198 (83%)	− 0.5	36 (15%)	**2.3**	5 (2%)	**− 2.9**	239
NMD	29 (91%)	1.1	0	**− 2.3**	3 (9%)	1.6	32
Total	273 (83.5%)		41 (12.5%)		13 (4%)		327

*N* numbers of patients; % percent; *p* probability value calculated by chi-square test of independence; *Cp* Pearson’s Contingency Coefficient C, Cp ≥ 0, values distant from 0 reflect some relationship, values approaching 1 correspond to a perfect association; *ASR* adjusted standardized residuals, values > 1.96 reflect a greater number, and those below < − 1.96 correspond to a smaller number than a random distribution.

In addition, the findings showed more/less common co-occurrence of as well as statistically significant relationships between *the size of the head—traditional classification (HC), as follows*:

- entities/syndromes associated with neurodysfunction (Table 3A). Normal head size rarely co-occurred with ACM and muscular dystrophy limb girdle (LGMD). Microcephaly was rarely found to co-occur with the state following surgery due to lumbar myelomeningocele and hydrocephalus (sasMMC&HCP) and frequently with Down syndrome (DS). Macrocephaly rarely found to co-occur with CP and frequently with the state following surgery due to lumbar myelomeningocele (sasMMC), ACM, HCP, PMS, and LGMD. The relationship was statistically significant (p < 0.001),
- classification with regard to etiopathogenesis, presence and character of encephalopathy (Table 3B and Fig. 1B). Microcephaly frequently co-occurred in children with GD and rarely in those with NMD. Macrocephaly frequently co-occurred with NTDs or NMD and rarely with CP. The relationship was statistically significant (p < 0.001),
- classification with regard to the presence and character of encephalopathy (Table 4B and Fig. 1D). Microcephaly frequently occurred in NPE and rarely in the subgroup with NMD. Macrocephaly was found more often in the group with NMD and less frequently in the subgroup with NPE. The relationship was statistically significant (p = 0.006, Cp = 0.206),
- encephalopathy in neural tube defects (Table 4C and Fig. 2A). Normal head size was often found in sasMMC&HCP and rarely in sasMMC, a state following surgery due to parieto-occipital meningocele (sasMM), ACM, and HCP. Microcephaly co-occurred frequently with sasMMC, sasMM, ACM, HCP but rarely with sasMMC&HCP. The relationship was statistically significant (p = 0.004, Cp = 0.564),
- type of spasticity (Table 4D and Fig. 2B). Normal head size was more frequent in children with hemiplegia. Microcephaly was more often associated with tetraplegia. The relationship was statistically significant (p = 0.034, Cp = 0.211),
- type of spasticity (Table 4E and Fig. 2C). Normal head size was rarely found to co-occur with tetraplegia. Microcephaly more often accompanied tetraplegia. The relationship was statistically significant (p = 0.034; Cp = 0.211),
- epilepsy (Table 4G and Fig. 2E). Normal head circumference rarely co-occurred with epilepsy and frequently with a lack of epilepsy. Microcephaly often co-occurred with epilepsy and rarely when epilepsy was not present. The relationship was statistically significant (p < 0.001),
- hypothyroidism (Table 5B and Fig. 3B). Normal head circumference frequently co-occurred in the absence of hypothyroidism and was rarely found in patients with hypothyroidism. Microcephaly frequently co-occurred with hypothyroidism, and in the absence of this condition, it rarely was found. The relationship was statistically significant (p < 0.001).

**Table 3:** The size of the head—traditional classification (HC) and (A) units and syndromes associated with neurodysfunction, (B) classification with regard to the etiopathogenesis, presence and character of encephalopathy.

(A) The size of the head—traditional classification (HC) and entities and syndromes associated with neurodysfunction							
Entities and syndromes associated with neurodysfunction	The size of the head—traditional classification (HC) by z-score HC (*p* < 0.001; Cp = 0.509)						
	Normal		Microcephaly		Macrocephaly		Total
	N (%)	ASR	N (%)	ASR	N (%)	ASR	N (%)
NBIA-MPAN	2	1.0	0	− 0.8	0	− 0.5	2 (100.0%)
GSD II	1	0.7	0	− 0.5	0	− 0.3	1
LCHAD	1	0.7	0	− 0.5	0	− 0.3	1
SLO	0	− 1.5	1	1.9	0	− 0.3	1
GLUT1d	1	0.7	0	− 0.5	0	− 0.3	1
NKH	1	0.7	0	− 0.5	0	− 0.3	1
SMEI	1	0.7	0	− 0.5	0	− 0.3	1
sasMMC&HCP	14 (82%)	1.3	0	− 2.3	3 (18%)	1.2	17
sasMMC	1 (33%)	− 1.3	0	− 0.9	2 (67%)	3.4	3
sasMM	0	− 1.5	1	1.9	0	− 0.3	1
ACM	0	− 2.1	1 (50%)	1.0	1 (50%)	2.0	2
HCP	0	− 1.5	0	− 0.5	1	3.1	1
DS	5 (45.5%)	− 1.7	6 (54.5%)	2.6	0	− 1.1	11
ES	0	− 1.5	1	1.9	0	− 0.3	1
PMS	1 (50%)	− 0.6	0	− 0.8	1 (50%)	2.0	2
MWS	1	0.7	0	− 0.5	0	− 0.3	1
AS	0	− 1.5	1	1.9	0	− 0.3	1
DGS	0	− 1.5	1	1.9	0	− 0.3	1
46,XY,del(X)(q24)	1	0.7	0	− 0.5	0	− 0.3	1
CdLS	1	0.7	0	− 0.5	0	− 0.3	1
SDS	1	0.7	0	− 0.5	0	− 0.3	1
PWS	1	0.7	0	− 0.5	0	− 0.3	1
46 XX, add(2)(q25)	1	0.7	0	− 0.5	0	− 0.3	1
46XX, del (12) (q24.21q24.23)	1	0.7	0	− 0.5	0	− 0.3	1
FAS	0	− 1.5	1	1.9	0	− 0.3	1
CP	167 (70%)	0.9	57 (24%)	1.3	15 (6%)	− 3.3	239
HMSN	7 (87.5%)	1.2	0	− 1.5	1 (12.5%)	0.3	8
LGMD	2 (29%)	− 2.3	0	− 1.4	5 (71%)	5.7	7
BMD	2 (67%)	− 0.1	0	− 0.9	1 (33%)	1.4	3
DMD	6 (86%)	1.0	0	− 1.4	1 (14%)	0.4	7
TD	1	0.7	0	− 0.5	0	− 0.3	1
AMC&N	2 (67%)	− 0.1	1 (33%)	0.5	0	− 0.6	3
CM	1	0.7	0	− 0.5	0	− 0.3	1
SMA	1 (50%)	− 0.6	1 (50%)	1.0	0	− 0.5	2
Total	224 (68.5%)		72 (22.0%)		31 (9.5%)		327

(B) The size of the head—traditional classification (HC) and classification with regard to the etiopathogenesis, presence and character of encephalopathy							
Classification with regard to the etiopathogenesis, presence and character of encephalopathy	The size of the head—traditional classification (HC) by z-score HC (*p* < 0.001; Cp = 0.315)						
	Normal		Microcephaly		Macrocephaly		Total
	N (%)	ASR	N (%)	ASR	N (%)	ASR	N (%)
E-MD	6 (86%)	1.0	1 (14%)	− 0.5	0	− 0.9	7
EE	1	0.7	0	− 0.5	0	− 0.3	1
NTDs	15 (62.5%)	− 0.7	2 (8.5%)	− 1.7	7 (29%)	− 3.4	24
GD	13 (56.5%)	− 1.3	9 (39%)	2.1	1 (4.5%)	− 0.9	23
TE	0	− 1.5	1	1.9	0	− 0.3	1
CP	167 (70%)	0.9	57 (24%)	1.3	15 (6%)	− 3.3	239
NMD	22 (69%)	0.0	2 (6%)	− 2.3	8 (25%)	3.2	32
Total	224 (68.5%)		72 (22%)		31 (9.5%)		327

*N* numbers of patients; % percent; *p* probability value calculated by chi-square test of independence, *Cp* Pearson’s Contingency Coefficient C, Cp ≥ 0, values distant from 0 reflect some relationship, values approaching 1 correspond to a perfect association; *ASR* adjusted standardized residuals, values > 1.96 reflect a greater number, and those below < − 1.96 correspond to a smaller number than a random distribution.

**Table 4:** The size of the head and classification with regard to the presence and character of encephalopathy (A, B), neural tube defects (C), type of spasticity (D, E), and epilepsy (F, G).

(A) The size of the head—dysmorphology classification (HC) and classification with regard to the presence and character of encephalopathy							
Classification with regard to the presence and character of encephalopathy	The size of the head—dysmorphology classification (HC) by z-score HC (*p* = 0.060; Cp = 0.164)						
	Normal		Microcephaly		Macrocephaly		Total
	N (%)	ASR	N (%)	ASR	N (%)	ASR	N (%)
PE	8	1.3	0	− 1.1	0	− 0.6	8
NPE	236 (82%)	− 1.6	41 (14%)	2.6	10 (4%)	− 1.2	287
NMD	29 (91%)	1.1	0	− 2.3	3 (9%)	1.6	32
Total	273 (83.5%)		41 (12.5%)		13 (4%)		327

(B) The size of the head—traditional classification (HC) and classification with regard to the presence and character of encephalopathy							
Classification with regard to the presence and character of encephalopathy	The size of the head—traditional classification (HC) by z-score HC (*p* = 0.006; Cp = 0.206)						
	Normal		Microcephaly		Macrocephaly		Total
	N (%)	ASR	N (%)	ASR	N (%)	ASR	N (%)
PE	7 (87.5%)	1.2	1 (12.5%)	− 0.7	0	− 0.9	8
NPE	195 (68%)	− 0.6	69 (24%)	2.4	23 (8%)	− 2.4	287
NMD	22 (69%)	0.0	2 (6%)	− 2.3	8 (25%)	3.2	24
Total	224 (68.5%)		72 (22%)		31 (9.5%)		327

(C) Relationship between the size of the head—traditional classification (HC) and encephalopathy in neural tube defects							
Encephalopathy in neural tube defects	The size of the head—traditional classification (HC) by z-score HC (*p* = 0.004; Cp = 0.564)						
	Normal		Microcephaly		Macrocephaly		Total
	N (%)	ASR	N (%)	ASR	N (%)	ASR	N (%)
sasMMC&HCP	14 (82%)	3.1	0	− 2.3	3 (18%)	− 1.9	17
sasMMC, sasMM, ACM, HCP	1 (14%)	− 3.1	2 (29%)	2.3	4 (57%)	1.9	7
Total	15 (63%)		2 (8%)		7 (29%)		24

(D) The size of the head—traditional classification (HC) and type of spasticity							
Type of spasticity	The size of the head—traditional classification (HC) by z-score HC (*p* = 0.034; Cp = 0.211)						
	Normal		Microcephaly		Macrocephaly		Total
	N (%)	ASR	N (%)	ASR	N (%)	ASR	N (%)
Diplegia	62 (69%)	− 0.4	19 (21%)	− 0.6	9 (10%)	1.9	90
Hemiplegia	47 (82.5%)	2.3	8 (14%)	− 1.9	2 (3.5%)	− 1.0	57
Tetraplegia	48 (63%)	− 1.7	25 (33%)	2.4	3 (4%)	− 1.0	76
Total	157 (71%)		52 (23%)		14 (6%)		223

(E) Relationship between the size of the head—traditional classification (HC) and type of spasticity							
Type of spasticity	The size of the head—traditional classification (HC) by z-score HC (*p* = 0.041; Cp = 0.167)						
	Normal		Microcephaly		Macrocephaly		Total
	N (%)	ASR	N (%)	ASR	N (%)	ASR	N (%)
Tetraplegic	48 (63%)	− 1.7	25 (33%)	2.4	3 (4%)	− 1.0	76
Other: diplegic, hemiplegic	109 (74%)	1.7	27 (18%)	− 2.4	11 (8%)	1.0	147
Total	157 (70%)		52 (23%)		14 (7%)		223

(F) The size of the head—dysmorphology classification (HC) and epilepsy							
Additional diagnosis epilepsy	The size of the head—dysmorphology classification (HC) by z-score HC (*p* = 0.000; Cp = 0.251)						
	Normal		Microcephaly		Macrocephaly		Total
	N (%)	ASR	N (%)	ASR	N (%)	ASR	N (%)
Present	59 (69%)	− 4.3	23 (27%)	4.6	4 (4%)	0.4	86
Absent	214 (89%)	4.3	18 (7%)	− 4.6	9 (4%)	− 0.4	241
Total	273 (83.5%)		41 (12.5%)		13 (4%)		327

(G) The size of the head—traditional classification (HC) and epilepsy							
Additional diagnosis epilepsy	The size of the head—traditional classification (HC) by z-score HC (*p* < 0.001; Cp = 0.245)						
	Normal		Microcephaly		Macrocephaly		Total
	N (%)	ASR	N (%)	ASR	N (%)	ASR	N (%)
Present	45 (52%)	− 3.8	34 (40%)	4.6	7 (8%)	− 0.5	86
Absent	179 (74%)	3.8	38 (16%)	− 4.6	24 (10%)	0.5	241
Total	224 (68.5%)		72 (22%)		31 (9.5%)		327

*N* numbers of patients; % percent; *p* probability value calculated by chi-square test of independence; *Cp* Pearson’s Contingency Coefficient C, Cp ≥ 0, values distant from 0 reflect some relationship; values approaching 1 correspond to a perfect association; *ASR* adjusted standardized residuals, values > 1.96 reflect a greater number, and those below < − 1.96 correspond to a smaller number than a random distribution.

**Table 5:** The size of the head, hypothyroidism, epilepsy, type of spasticity.

(A) The size of the head—dysmorphology classification (HC) and hypothyroidism							
Additional diagnosis hypothyroidism	The size of the head—dysmorphology classification (HC) by z-score HC (*p* = 0.024; Cp = 0.150)						
	Normal		Microcephaly		Macrocephaly		Total
	N (%)	ASR	N (%)	ASR	N (%)	ASR	N (%)
Present	9 (64%)	− 2.0	5 (36%)	2.7	0	− 0.8	14
Absent	264 (84.5%)	2.0	36 (11.5%)	− 2.7	13 (4%)	0.8	313
Total	273 (83.5%)		41 (12.5%)		13 (4%)		327

(B) The size of the head—traditional classification (HC) and hypothyroidism							
Additional diagnosis hypothyroidism	The size of the head—traditional classification (HC) by z-score HC (*p* < 0.001; Cp = 0.213)						
	Normal		Microcephaly		Macrocephaly		Total
	N (%)	ASR	N (%)	ASR	N (%)	ASR	N (%)
Present	5 (36%)	− 2.7	9 (64%)	3.9	0	− 1.2	14
Absent	219 (70%)	2.7	63 (20%)	− 3.9	31 (10%)	1.2	313
Total	224 (68.5%)		72 (22%)		31 (9.5%)		327

(C) Relationship between epilepsy and type of spasticity							
Additional diagnosis epilepsy	Type of spasticity (*p* < 0.001; Cp = 0.257)						Total
	Tetraplegia		Diplegia		Hemiplegia		
	N (%)	ASR	N (%)	ASR	N (%)	ASR	N (%)
Present	33 (52%)	3.6	14 (22%)	− 3.5	16 (26%)	0.0	63
Absent	43 (27%)	− 3.6	76 (47%)	3.5	41 (26%)	0.0	160
Total	76 (34%)		90 (40%)		57 (26%)		223

(D) Relationship between type of spasticity and z-score HC							
Nominal regression	Quantitative dependent variable z-score HC						
**Qualitative dependent variable type of spasticity**							
Tetraplegia (34%)	< 0.001	p					
	0.782 0.677–0.904	OR					
Other: diplegia, hemiplegia (66%)	Reference group						

(E) Relationship between hypothyroidism and z-score HC							
Nominal regression	Quantitative dependent variable z-score HC						
**Qualitative dependent variable additional diagnosis**							
Presence of hypothyroidism (4.5%)	< 0.001	p					
	0.599 0.457–0.785	OR					
Lack of hypothyroidism (95.7%)	Reference group						

(F) Relationship between epilepsy and z-score HC							
Nominal regression	Quantitative dependent variable z-score HC						
**Qualitative dependent variable additional diagnosis**							
Presence of epilepsy (26%)	< 0.001	p					
	0.804 0.711–0.909	OR					
Lack of epilepsy (74%)	Reference group						

*N* numbers of patients; % percent; *p* probability value calculated by chi-square test of independence; *Cp* Pearson’s Contingency Coefficient C, Cp ≥ 0, values distant from 0 reflect some relationship; values approaching 1 correspond to a perfect association; *ASR* Adjusted standardized residuals, values > 1.96 reflect a greater number, and those below < − 1.96 correspond to a smaller number than a random distribution; *OR* odds ratio (95% confidence interval).

Cp is higher for the traditional classification:

- amounting to 0.509 compared to the dysmorphology criterion of 0.465 (Tables 2A, 3A). The traditional criterion in this case more effectively differentiated the relationship between head size and specific medical conditions,
- amounting to 0.315 compared to the dysmorphology criterion of 0.273 (Tables 2B, 3B). The traditional classification in this case more effectively differentiated the relationship between head size and the seven subgroups distinguished based on classification considering etiopathogenesis, as well as the occurrence and character of encephalopathy,
- amounting to 0.206 compared to the dysmorphology criterion of 0.146 (Table 4A,B). The traditional classification in this case more effectively differentiated the relationship between head size and the three subgroups distinguished based on classification taking into account the occurrence and character of encephalopathy,
- amounting to 0.213 compared to the dysmorphology criterion of 0.150 (Table 5A,B). The criterion of ± 2SD in this case more effectively differentiated the relationship between head size and hypothyroidism.

Cp was higher for dysmorphology than for traditional classification, at 0.251 and 0.245, respectively (Table 4F,G). The criterion of ± 3 SD in this case more effectively differentiated the relationship between head size and epilepsy.

Further analyses focused on the relationships between the quantitative independent variable, i.e., z-score HC, and a dependent qualitative variables such as divisions within subgroups or the presence of an additional diagnosis. Significant effects were found in the following cases: intragroup differences related to the spastic type, tetraplegia versus other, i.e., diplegia, hemiplegia (Table 5D); intragroup differences related to additional diagnoses, i.e., presence/absence of hypothyroidism (Table 5E); and presence/absence of epilepsy (Table 5F). In the first case, (Table 5D), higher z-score HC values correspond to a lower probability of co-occurring tetraplegia (p < 0.001). The likelihood of tetraplegia decreased with higher z-score HC (OR = 0.782). In the second case (Table 5E), higher z-score HC values corresponded to a lower probability of co-occurring hypothyroidism (p < 0.001). With a higher z-score HC, the likelihood of hypothyroidism decreased (OR = 0.599). In the third case (Table 5F), greater values of z-score HC reflect a lower likelihood of cooccurring epilepsy (p < 0.001); i.e., the higher the z-score HC value was, the lower the likelihood of cooccurring epilepsy was (OR = 0.801).

Relationships between additional diagnoses and tetraplegia were also examined, and epilepsy frequently co-occurred with tetraplegia (p < 0.001) (Table 5C and Fig. 3C).

## Discussion

Developmental defects of the cranium and the brain may be clinically isolated or occur as part of complex syndromes associated with other neurodevelopmental challenges, brain defects and abnormal body growth^21^. Research has shown that macrocephaly and microcephaly remain poorly defined and that a uniform diagnostic approach is urgently needed^21–29^. For the purposes of this article, the definitions of microcephaly and macrocephaly, including growth disorders, were used^4–6^. As disturbances in the differentiation of body proportions were not taken into account, relative or absolute microcephaly/macrocephaly was not distinguished. There is a relationship between the size of the brain and the size of the skull^30^, the body size^31^, and the neurological capacity of organisms^31^. Previous studies have shown that tetraplegia is accompanied by short stature^3^, though current studies have shown that tetraplegia is accompanied by microcephaly, which confirms the theory of encephalysis. Therefore, tetraplegia was not excluded from the analysis of relative microcephaly/macrocephaly; indeed, a proportional reduction in body size may be associated with a reduced brain size^31^. Definitive etiological diagnosis is crucial for predicting outcomes and for designing treatments administered to affected patients^21–29^, and this observation provided motivation for the current study. Another reason was the fact that, to our knowledge, no studies have investigated relationships between co-occurring abnormal cranium development and diseases or syndromes linked to neurodysfunction, also grouped according to the presented classification^1,3^. Hence, this study is the first to offer scientific evidence related to this issue.

According to Pirozzi et al.^21^, neurodevelopmental impairments linked to abnormal growth of the brain, with or without cortical defects, significantly contribute to morbidity and mortality and are associated with numerous consequences of a neurodevelopmental nature that manifest in infancy or early childhood^21^. The majority of children experiencing the diseases and syndromes discussed also had encephalopathy, indicating its prevalence. Encephalopathies are all conditions affecting brain structure. It is possible to distinguish various types of encephalopathy depending on the patient’s age and causes of the condition^32,33^. By taking into account the characteristics and expected presence of encephalopathy, the current study identified patients with progressive and nonprogressive encephalopathy. Less than 10% of the children in the study group were affected by neuromuscular disorders. Given that encephalopathies comprise disorders of hereditary or nonhereditary nature, i.e., congenital or acquired conditions that are primarily characterized by brain damage^32,33^ and may affect head size, it seems justified that the present findings show significant correlations of head size in both assessment systems (dysmorphology versus traditional) to patient classification based on the etiopathogenesis and presence and character of encephalopathy.

The use of a classification system differentiating the size of the head based on dysmorphological criteria allowed us to uncover the common cooccurrence of (as well as statistically significant relationships between) microcephaly and NPE (classification based on the presence and character of encephalopathy), CP (classification based on the etiopathogenesis, presence and character of encephalopathy), ES, DGS, and CP (entities/syndromes associated with neurodysfunction). Macrocephaly is frequently found with NTDs (classification based on the etiopathogenesis, presence and character of encephalopathy), ACM, HCP, PMS, and BMD (entities/syndromes associated with neurodysfunction). Microcephaly rarely cooccurs in children with NMD and macrocephaly with CP. The definition of microcephaly^6,34^ and macrocephaly^35^ based on dysmorphological criteria is used by geneticists^6,34,35^, and it has been used for studies on the etiopathogenesis of microcephaly primary hereditary. Genes responsible for microcephaly primary hereditary implicate a wide variety of molecular and cellular mechanisms involved in the regulation of cerebral cortical size during development. Studying these syndromes can also reveal molecular mechanisms that are critical for the regulation of brain size and human brain evolution^6^. Primary (of prenatal onset) or secondary (of postnatal onset) microcephaly results from an imbalance between progenitor cell production and cell death that leads to a reduced number of neuronal and glial cells within the brain, resulting in reduced brain growth. Secondary microcephaly is associated with increased neuronal death and can be associated with serine deficiency or thiamine pyrophosphate transporter deficiency and metabolic diseases associated with progressive encephalopathy^34^. For example, macrocephaly (criterion > 3 SD) occurs in megalencephaly-polymicrogyria-polydactyly hydrocephalus syndrome^35^.

The use of a classification system that differentiates the size of the head based on traditional criteria allowed us to show that microcephaly frequently occurs in NPE (classification based on the presence and character of encephalopathy), in children and adolescents with GD (classification based on the etiopathogenesis, presence and character of encephalopathy), and in DS (entities/syndromes associated with neurodysfunction). Macrocephaly was found more often in the group with NMD (classification with regard to the presence and character of encephalopathy), NTDs, or NMD (classification with regard to the etiopathogenesis, presence and character of encephalopathy) and was frequently found to co-occur with sasMMC, ACM, HCP, PMS, and LGMD (entities/syndromes associated with neurodysfunction). Conversely, microcephaly rarely co-occurred in children with NMD and macrocephaly with CP, results that were significant. The definition of microcephaly^4,5^ and macrocephaly^36–38^ based on traditional criteria is used by clinicians, who often use magnetic resonance imaging^4,5,36–38^, microbiological^5,38^ and genetic tests^4,5,36,37^ in the differential diagnostic process. Our research confirms the utility of genetic tests for diagnostic and differential processes.

The advantage of the present study is the fact that its findings provide an unambiguous answer to the question formulated in the purpose of the study, namely, which of the two existing criteria used in assessing cranial development defects—the system applied in dysmorphology and the one traditionally used in clinical practice—should be employed in daily work. The presented evidence suggests greater effectiveness of the traditional classification, as it enabled the identification of more relationships. Moreover, the traditional classification more effectively differentiated relationships between head size and all the subgroups distinguished based on the system taking into account the etiopathogenesis as well as the presence and character of encephalopathy.

Different definitions of microcephaly are used in studies examining the relationship between microcephaly and epilepsy: HC < 3percentile^22^, HC < 2SD^23,24^, HC < 3SD^25^. The current study shows that in children and adolescents with diseases or syndromes linked to neurodysfunction, microcephaly co-occurred with epilepsy as well as with hypothyroidism. Von der Hagen et al.^22^ carried out a retrospective study in a group of 680 children with microcephaly. The causes of this condition were identified in 59% of the children, whereas no definite diagnosis was formulated for the remaining 41%. In the former group, genetic causes were identified in approximately half of the patients; perinatal and postnatal brain damage accounted for 45% and 3% of the cases, respectively. In the study by Von der Hagen et al., epilepsy was diagnosed in 43% of children with microcephaly^22^. Similar results were reported by Abdel-Salam et al.^23^. Although their study involved a much smaller group, i.e., only 66 children with microcephaly, the authors also identified epilepsy in 40.9% of them^23^. Furthermore, it has been reported that epilepsy is more common in children with secondary microcephaly than in those with primary microcephaly^23,24^. Similarly, Ashwal et al.^25^ pointed out that microcephaly is associated with comorbidities such as epilepsy^25^. In our study, the criterion of ± 3 SD more effectively differentiated the relationship between head size and epilepsy.

Another finding of the current study is related to the co-occurrence of microcephaly and hypothyroidism in children and adolescents with diseases or syndromes associated with neurodysfunction. Research has shown that normal thyroid metabolism is necessary for human development, including the formation and functioning of the brain. However, abnormal thyroid metabolism is increasingly diagnosed in the spectrum of pediatric neurological disorders^39^. Moreover, in their literature review investigating neurological symptoms of impaired thyroid metabolism, Kurian and Jungbluth^39^ also point out the correlation between hypothyroidism and microcephaly^39^. A similar relationship was reported by Carré et al.^40^. Nonetheless, the criteria for the diagnosis of microcephaly are often not included In reviews of the literature considering the relationship between microcephaly, cerebral abnormalities and hypothyroidism^39,40^. In our study, the criterion of ± 2 SD effectively differentiated the relationship between head size and hypothyroidism.

The current study also shows that microcephaly more commonly co-occurs with tetraplegia linked to CP and that the former is often found to exist simultaneously with epilepsy. The occurrence of microcephaly (criterion: HC < 3 SD) in patients with spastic quadriplegia was reported by Cavallin et al.^41^, and Singhi and Sain^42^ reported microcephaly (criterion: HC < 2SD) in 64.27% of children with CP in North India^42^, and microcephaly is the most frequent type being spastic quadriplegia^42,43^. Hadjipanayis et al.^44^ carried out a study involving 323 patients with CP palsy and observed that epilepsy occurred in this group at a rate of 41.8% and in nearly one in two patients with spastic tetraplegia^44^. Reports that emphasize the fact that subjects with epilepsy are more at risk of accidental injuries than are individuals who do not experience seizures can be found in the literature. The most common injuries include head contusions, which consequently may lead to spastic quadriplegia^45^.

Based on our retrospective analyses, we were able to systematize certain data, correlations and definitions^46^. Human developmental abnormalities such as microcephaly and macrocephaly are phenotypic abnormalities, and their definitions should be standardized and included in HPO terms^47,48^. We hope that the dependencies presented herein will contribute to the differential diagnostic process^4,10^ and to standardization of the definitions of microcephaly and macrocephaly.

### Limitations

The limitations of this study are as follows. (a) This study was retrospective in nature. However, to conduct such a retrospective analysis, studies on the secular development trend of children and adolescents in Rzeszow were carried out in 2013 and 2014 with the measurement of head circumference, and without this, the present analysis would not be possible. Another limitation is (b) the dominance of the male sex in the study group. Further prospective studies using a larger group of subjects are necessary.

## Conclusions

Children and adolescents with syndromes or diseases associated with neurodysfunction present abnormal cranial development (head size). Microcephaly rarely co-occurs in children with NMD; macrocephaly frequently co-occurs with NTDs or NMD but rarely with CP. Children and adolescents with syndromes or diseases associated with neurodysfunction have microcephaly co-occurring with epilepsy and hypothyroidism. It was shown that traditional classification enabled the identification of a greater number of relationships; therefore, it should be used in daily practice. Overall, there is a need for standardization of the definition of microcephaly and macrocephaly and for their inclusion in HPO terms.

## Abbreviations

HC: Head circumference
OFC: Occipitofrontal circumference
x − 3SD: Difference between the mean and 3 standard deviations
x + 3SD: Total of the mean and 3 standard deviations
x − 2SD: Difference between the mean and 2 standard deviations
x + 2SD: Total of the mean and 2 standard deviations
SD: Standard deviation
KRORE: Clinical Regional Rehabilitation and Education Centre
CP: Cerebral palsy
z-score HC: Z-Score for head circumference
ASR: Adjusted standardized residual
PE: Progressive encephalopathy
NPE: Nonprogressive encephalopathy
NMD: Neuromuscular disease
GD: Genetic disorder
$$\overline{x}$$: Arithmetic mean
Me: Median
Min: Minimum value
Max: Maximum value
c25: 25Th centile
c75: 75Th centile
HCP: Isolated hydrocephalus
ES: Edwards syndrome
DGS: Di George syndrome
ACM: Arnold–Chiari malformation
PMS: Phelan–McDermid syndrome
BMD: Becker’s muscular dystrophy
NTD: Neural tube defect
LGMD: Muscular dystrophy limb-girdle
sasMMC&HCP: State following surgery due to lumbar myelomeningocele and hydrocephalus
DS: Down syndrome
sasMMC: State following surgery due to lumbar myelomeningocele
sasMM: State following surgery due to parieto-occipital meningocele
HPO: Human Phenotype Ontology
